# Supplementary material for: Efficacy and safety of Mojeaga remedy in combination with conventional oral iron therapy for correcting anemia in obstetric population: A phase II randomized pilot clinical trial
Source: PLoS One. 2023 May 8;18(5):e0285474. doi: 10.1371/journal.pone.0285474 (PMC10166496; doi:10.1371/journal.pone.0285474)
Supplement: S1 File — (DOCX) [file pone.0285474.s002.docx]

**Efficacy and safety of Mojeaga^®^ in combination with conventional oral iron therapy for correcting anemia in gynecological and obstetrics patients: a pilot randomized clinical trial in NAUTH, Nnewi.**

**A PROPOSAL**

**SUBMITTED**

**BY**

**DR GEORGE UCHENNA ELEJE (MBBS, MSC, FMCOG, FWACS, FICS)**

SENIOR LECTURER AND HONORARY CONSULTANT

DEPARTMENT OF OBSTETRICS AND GYNAECOLOGY

NNAMDI AZIKIWE UNIVERSITY, AWKA/ NNAMDI AZIKIWE UNIVERSITY TEACHING HOSPITAL (NAUTH), NNEWI, SOUTH-EAST NIGERIA

**TO**

**NAUTH ETHICS COMMITTEE**

**FEBRUARY, 2019**

**THE DECLARATION**

This proposal is original and has not previously been submitted to any other authority for consideration. The purpose of this study is not to attract grant but for dissemination of scientific knowledge through publication with the prospective authors/ co-authors/collaborators/researchers in a reputable impact factor journal. The protocol will be shared publicly.

--------------------------------------------------------

**DR GEORGE .U. ELEJE, MBBS (Awka), FMCOG, FWACS, FICS**

(PRINCIPAL INVESTIGATOR I)

SENIOR LECTURER/ HONORARY CONSULTANT

DEPARTMENT OF OBSTETRICS AND GYNAECOLOGY, NNAMDI AZIKIWE UNIVERSITY TEACHING HOSPITAL, NNEWI

**COLLABORATORS**

- **DR. IFEOMA C. AJUBA [Haematologist]**
- **PROF. JOSEPH .I. IKECHEBELU [Obstetrician-Gynecologist]**
- **DR. EJEATULUCHUKWU OBI [Pharmacology and Therapeutics & Toxicologist]**
- **DR. CHIGOZIE .G. OKAFOR [Obstetrician-Gynecologist]**
- **DR CHIDOZIE GODWIN UGOCHUKWU [Biochemist]**
- **DR CHUKWUEMEKA OKORO [Obstetrician-Gynecologist]**
- **MR CHIKA ASUOHA [Chemical Pathologist]**
- **DR RICHARD EGEONU [Obstetrician-Gynecologist]**

**ABSTRACT**

**BACKGROUND**

Mojeaga^®^ (a special blend of *Alchornea, Pennisetum, and Sorghum extracts*) may be very effective in correcting anemia in gynecological and obstetrics patients. Limited available studies in patients suggest a seemingly high efficacy but provide little information on benefits and adverse events. Study on its use in treatment of iron deficiency anemia in adult patients who are intolerant to oral iron preparations or who refuse blood transfusion is highly desirable. To our knowledge, there is no prior randomized trial on the effectiveness of Mojeaga when co-administered with oral iron therapy schedule for correction of anemia in Obstetrics and gynecology practice.

**OBJECTIVES**

To determine the efficacy, safety and tolerability of Mojeaga as adjunct to conventional oral iron therapy for correction of anemia in puerperal and obstetrics patients.

**METHODS**

A pilot open-label randomized clinical trial will be conducted in patients with confirmed diagnosis of anemia (<10.0 g/dl) in Obstetrics and gynecological Clinics of Nnamdi Azikiwe University Teaching Hospital, Nnewi. All laboratory parameters will be carried out at Nnamdi Azikiwe University Teaching Hospital, Nnewi, laboratory and other collaborating hospitals by Dr Ifeoma Ajuba and Chika Asuoha. Eligible patients will be randomized to either Mojeaga 50 mls to be administered three times daily in conjunction with iron therapy Astyfer one capsule two times daily for 2 weeks or conventional iron therapy alone (Astyfer one capsule two times daily) without Mojeaga for 2 weeks. Repeat hemoglobin/hematocrit level will be done 2 weeks post-initial therapy. Primary outcome measures will be changes in the hematocrit level at two weeks after initial therapy and mean or median hematocrit level at two weeks after initial therapy. Secondary outcome measures will include incidence of disappearance of any previously existing anemic symptoms, and presence and incidence of any adverse events. Analysis will be by intention-to-treat.

#### TRIAL REGISTRATION: Pan Africa Clinical Trial Registry PACTR201901852059636.

**INTRODUCTION**

Anemia in women is a major public health burden worldwide, particularly in low- and middle-income countries, including Nigeria [1]. According to the World Health Organization, anemia affects approximately 2 billion people worldwide, which results in an estimated global prevalence of almost 25% [2]. The World Health Organization (WHO) estimates the prevalence of anemia among pregnant women to vary from 53.8% to 90.2% in developing countries, and 8.3–23% in developed countries.

Anemia impacts upon more than half the preschool children and pregnant women in developing countries and at least 30%–40% in developed countries [2]. Additionally, a high prevalence of anemia is associated with the elderly and with acute as well as chronic conditions. The World Health Assembly has set a target of a 50% reduction in anemia among women of reproductive age by 2025 [1].

Although several factors such as vitamin deficiencies (A, B12, folic acid), infectious diseases (parasites), chronic inflammation, and genetic disorders may cause anemia, iron deficiency is undoubtedly the most common cause of anemia worldwide [3]. Notably, iron deficiency affects not only red blood cell production but also cellular functions related to muscle metabolism, mitochondrial function, neurotransmitters, DNA synthesis, and the immune system [4, 5]. For example, throughout pregnancy, total iron requirements increase to meet the major hematologic changes of the mother and the increasing demands of the growing fetus [6]. Iron deficiency anemia (IDA) during pregnancy is associated with an increased risk of preterm birth, low birthweight, fetal growth restriction, and increased neonatal and maternal mortality. Furthermore, iron deficiency may predispose a person to postpartum IDA, peripartum blood transfusion, infections, and precipitate heart failure [6].

IDA is often treated with oral iron supplement even in pregnancy. However, due to gastrointestinal side effects such as nausea, vomiting, and constipation, compliance is often poor and results in subsequent discontinuation [4, 6, 7]. As such, intravenous iron administration or blood transfusion is increasingly being recommended for women who are non-compliant with oral iron, have severe IDA, or those who require rapid intervention [6]. However, intravenous drug administration have been infrequently utilised in clinical practice due to undesirable adverse reactions, including severe allergic reactions and anaphylaxis. Blood transfusion is not without complications and some religious groups object to blood transfusions.

Mojeaga^®^ (produced by Mojeaga International Ventures Ltd, Nigeria) is a natural preparation containing a combined *Alchornea*, *Pennisetum*, and *Sorghum* extracts [8,9]. It contains free organic oral iron preparation among other nutrients that allows co-administration of conventional hematinics or other iron preparations. Mojeaga has been approved by the National Agency for Food and Drug Administration and Control (NAFDAC) with NAFDAC registration number of A7-0996L. The *Pennisetum* extract and *Sorghum* extract in Mojeaga are excellent sources of iron, potassium, flavonoids and other phytonutrients. According to the manufacturers, Mojeaga works by promoting healthy metabolic processes because it contains high levels of B-group vitamins [8, 9]. Mojeaga also works by effectively mopping up the free radicals and reactive oxygen species, and quickly reverse the lipid peroxidative and cellular damages. It increases the levels of packed cell volume and hemoglobin and contains antioxidants and natural minerals. It also has strong anti-inflammatory property and enhances general body metabolism. It builds immune system, and it is high in natural iron [8, 9]. There is an additional major benefit of the Mojeaga, in that it can be administered in relatively high normal doses in a short period of time [10].

In one recent report, anemic pregnant women of gestational age of 36 weeks with IDA of hemoglobin level of 5g/dl received Mojeaga in addition with oral iron therapy (adjunct therapy) for two weeks, and had their hemoglobin appreciated to 10 g/dl two weeks after treatment without any adverse pregnancy outcome or neonatal outcome [8]. In other published reports, significantly higher number of women achieved anemia correction within a shorter time frame, and there were markedly fewer gastrointestinal treatment-related adverse events especially when the drug is administered in sips [9, 10, 11]. This lends support to the hypothesis that Mojeaga in combination with oral iron therapy is superior to oral iron therapy alone for correcting anemia in gynecological and obstetrics patients.

To our knowledge, this is the first randomized study that will be reporting on efficacy and safety of combined Mojeaga and oral iron therapy schedule. Therefore, this proposed study is aimed at determining the effectiveness, safety and tolerability of Mojeaga as adjunct to conventional oral iron therapy for correcting anemia in gynecological and obstetrics patients.

**AIMS AND OBJECTIVES**

**AIM:** To determine the efficacy and safety of Mojeaga on puerperal and obstetrics patients with diagnosis of anemia, in NAUTH, Nnewi, south-eastern Nigeria.

**SPECIFIC OBJECTIVES**

- 1. To determine the mean hemoglobin/change in hematocrit level following combined Mojeaga and conventional iron therapy among puerperal and obstetrics patients diagnosed with anemia compared with that of those with conventional iron therapy alone.
  2. To determine the proportion of disappearance of anemic symptoms following combined Mojeaga and conventional iron therapy among puerperal and obstetric patients diagnosed with anemia compared with that of those with conventional iron therapy alone.
  3. To determine the proportion of maternal adverse events following combined Mojeaga and conventional iron therapy among puerperal and obstetric patients diagnosed with anemia compared with that of those with conventional iron therapy alone.

**OUTCOME MEASURES**

**PRIMARY**

1. Mean or median hematocrit level at two weeks after initial therapy.
2. Changes in the hematocrit level at two weeks after initial therapy.

**SECONDARY**

1. Proportion of patients with persisting anemic symptoms (weakness, easy fatigability) at two weeks after initial therapy.
2. Incidence of any maternal adverse events (such as diarrhea, nausea, vomiting and colitis) following commencement of therapy.
3. Incidence of any fetal adverse events (such as preterm labor or jaundice) following commencement of therapy.
4. Mean levels of renal function parameters (serum electrolyte, urea and creatinine) at two weeks after initial therapy.
5. Mean levels of liver function parameters (AST, ALT and ALP) and lipid profiles at two weeks after initial therapy.

**METHODS**

**Study setting**

The study will be carried out at the Obstetrics and gynecology clinics of Nnamdi Azikiwe University Teaching Hospital (NAUTH), Nnewi, a tertiary hospital in the South-east Nigeria, as well as other collaborating hospitals in south-east Nigeria.

**Study design**

This will be an open-label randomized clinical trial.

**Study population**

The participants will comprise both obstetrics and puerperal adult patients with clinical and laboratory diagnosis of anemia and who will be give written informed consent before recruitment. The patients will be recruited at the outpatient clinic or antenatal clinics or referred for treatment of their anemia, in the hospital.

**Inclusion criteria**

Both obstetrics and puerperal adult patients with confirmed clinical and laboratory diagnosis of anemia (<10.0 g/dl) and with normal liver and renal function markers or profiles will be included.

**Exclusion criteria**

- Pregnant women at first trimester of pregnancy (because of nausea and vomiting at this period).
- Multiple gestation
- Chronic medical disorders including HIV/AIDS, Cancers, etc.
- Women on chronic medications that cause anemia

**Randomization and Allocation Sequence**

Following consent, patients at the selected hospitals will be screened for eligibility. The patients, eligible for the study, will be randomized into two groups using simple (block) randomisation using a radomisation table to be created by a computer software program by a person not involved in the study and available at [**http://mahmoodsaghaei.tripod.com/Softwares/randalloc.html**](http://mahmoodsaghaei.tripod.com/Softwares/randalloc.html). Allocation sequences and codes will be concealed from the person allocating the participants to the intervention arms using numbered containers containing the drugs.

**Blinding of participants, Personnel and Outcome assessors**

Only the outcome assessors will be blinded (open-label trial).

**Study Procedure/Drug Administration**

Patients with a clinical and laboratory diagnosis of anemia presenting in Outpatient Clinic or antenatal clinic of NAUTH Nnewi for symptoms or signs of anemia will be screened consecutively. All patients will also undergo routine medical examination including pulse rate, body weight, blood pressure determination and general examination to ascertain the presence of and severity of anemia. All consenting patients will be diagnosed to have either anemia or not after undergoing hematological test. Only patients with confirmed clinical and laboratory diagnosis of anemia will be randomized. Eligible patients will be sequentially allocated using an opaque sealed envelope to receive either Mojeaga and conventional oral Therapy, or the conventional oral iron Therapy alone. Therapies will be giving for two weeks**.**

**Intervention Therapy**

## Standard doses of 50 mls of Mojeaga will be administered three time daily in conjunction with iron therapy Astyfer (Til Healthcare PVT Ltd, Andhra Pradesh, 517588 India) capsules two times a day (breakfast and dinner) for 2 weeks.

**Control Therapy**

Standard doses of one capsule of Astyfer will be administered alone two times a day (breakfast and dinner) for 2 weeks. These patients with confirmed diagnosis of anemia will be on conventional iron therapy administered without Mojeaga.

**Follow-Up**

The trial is expected to last for at least 16 months. Recruitment will be within the first 6 months of commencement of the study and the follow up will be for at least 10 months from recruitment (ie total of 16 (6+10) months). All participants will be followed up in outpatient settings. During each follow-up weekly visit, patients will be contacted on phone on weekly basis to assess level of compliance. The patients will also be encouraged to record any side effects or adverse events in a paper that will be reviewed at each follow-up visit, and they will be explicitly asked about such events during each interview. The drug compliance will be checked before follow-up test. Any patient found to be developing complications such as worsening of the

symptoms from the study will be given appropriate treatment. Repeat hemoglobin level will be done 2 weeks post initiation of treatment in all the participants to confirm success of treatment (correction of anemia and levels of hemoglobin/hematocrit levels) and absence of adverse effects. All the pre and post (repeat) hemoglobin/hematocrit level will be carried out by hematologist: Dr Ifeoma C. Ajuba, while the LFT, Lipid profiles and SEUCR will be carried out by the Senior laboratory Scientist of Chemical Pathology- Chika Asuoha in Nnamdi Azikiwe University Teaching Hospital, Nnewi laboratory.

**Sample size determination**

## Since this is a pilot study, we assumed a mean hematocrit level of 29.1% in the control group, mean hematocrit level of 31.5% in the intervention group after the intervention, 80% power, 95% confidence interval, standard deviation (SD) of 2.5, and a 15% dropout rate. Based on the assumptions, the minimum sample size required was 94 participants (47 in each arm). Furthermore, this sample size had 97% power to detect a change in hematocrit level from baseline (control: 6.5%; intervention: 10.5; SD: 3.8) between arms.

**Sampling Approach**

The randomized clinical trials are usually based on convenience sampling. After sampling, participants were randomly allocated to the intervention or control group (randomization) of the eligible, consenting women with diagnosis of anemia.

**Statistical Analysis**

Interim analysis will be planned. Data will be analysed using statistical analysis which will be performed by SPSS version 23, IBM Company, USA. The data will be expressed as the number (percentage), mean (standard deviation [SD]), and mean (95% confidence interval [95% CI] as appropriate. Categorical variables will be compared using the Chi-squared test and Fisher’s exact test, as necessary and relationship expressed using relative risks and 95% confidence intervals. Independent t-test will be used to compare mean of continuous variables between treatment groups. The intention-to-treat efficacy analyses will be based on all the patients who received the study medication and had completed the follow-up visit. The number needed to treat (NNT) analysis (the number of patients that need to be treated for one of them to benefit compared with a control in a clinical trial) will also done. Interim analyses will be planned after 30 participants have been recruited. Patients with no observed outcome will be considered as treatment failures. When possible, subgroup analysis will also be done. A p value ≤ 0.05 will be considered to be significant. Interim analyses of principal safety and effectiveness outcomes were performed on behalf of the data and safety monitoring committee by the trial statistician (who remained unaware of the treatment assignments) on two occasions.

**Ethical consideration**

The study will adhere to CONSORT guidelines. The study protocol will be approved by the Nnamdi Azikiwe University Teaching Hospital, Nnewi, Nigeria ethics committee. The study has been registered and approved by the Pan African Clinical trial registry: at [**www.pactr.org**](http://www.pactr.org/). The approval number is **PACTR201901852059636 (see the approval enclosed).** The procedures to be followed will be in accordance with the guidelines of the World Medical Association’s Declaration of Helsinki (1964, and its later amendments). The drug (Mojeaga) is registered with NAFDAC Nigeria with registration number of **NRN: A7-0996L**. The nature of the study will be carefully explained to the patients and their informed consent will be obtained before being recruited into the study. The rights of the patients to participate or withdraw from the study will be fully honored without any adverse consequence to the patient.

**Beneficence to participants**

No patient will make any form of payment for participation in this study. The results obtained in this pilot study will show supposed benefits of Mojeaga based on the manufacturers’ claims and lay the framework for a full-blown trial on these preparations.

**Non maleficence to participants**

There is insufficient data (except those from the manufacturer) to guarantee absence of any undesired effect.

**Incentives to patients**

The cost of the research will be entirely borne by the investigators. The subjects and the investigators will not be paid. No payment will be made to the subjects.

**Certification of Analysis**:

To ensure a high quality standardized formulations, the raw material was authenticated and the product was laboratory tested and certified by Prof JU Iyasele of Chemistry Department, University of Benin in accordance with Institute of Public analyst of Nigeria Decree no 100 of 1992 (see the certification enclosed).

**Funding:**

The research will partly be funded by the manufacturer of Mojeaga (Mojeaga International Ventures Ltd, Nigeria) and the researchers. The manufacturer will only be responsible for supplying the Mojeaga and Astyfer therapy to be used for the study. They will only supply drugs. The researchers will fund other expenses of the trial.

**REFERENCES**

1. [Heidkamp R](https://www.ncbi.nlm.nih.gov/pubmed/?term=Heidkamp%20R%5BAuthor%5D&cauthor=true&cauthor_uid=28904114), [Guida R](https://www.ncbi.nlm.nih.gov/pubmed/?term=Guida%20R%5BAuthor%5D&cauthor=true&cauthor_uid=28904114), [Phillips E](https://www.ncbi.nlm.nih.gov/pubmed/?term=Phillips%20E%5BAuthor%5D&cauthor=true&cauthor_uid=28904114), [Clermont A](https://www.ncbi.nlm.nih.gov/pubmed/?term=Clermont%20A%5BAuthor%5D&cauthor=true&cauthor_uid=28904114). The Lives Saved Tool (LiST) as a Model for Prevention of Anemia in Women of Reproductive Age. [J Nutr.](https://www.ncbi.nlm.nih.gov/pubmed/28904114) 2017 Nov;147(11):2156S-2162S. doi: 10.3945/jn.117.252429. Epub 2017 Sep 13.
2. [Toblli JE](https://www.ncbi.nlm.nih.gov/pubmed/?term=Toblli%20JE%5BAuthor%5D&cauthor=true&cauthor_uid=25525337), [Angerosa M](https://www.ncbi.nlm.nih.gov/pubmed/?term=Angerosa%20M%5BAuthor%5D&cauthor=true&cauthor_uid=25525337). Optimizing iron delivery in the management of anemia: patient considerations and the role of ferric carboxymaltose. [Drug Des Devel Ther.](https://www.ncbi.nlm.nih.gov/pubmed/25525337) 2014 Dec 11;8:2475-91. doi: 10.2147/DDDT.S55499. eCollection 2014.
3. [Pereira DIA](https://www.ncbi.nlm.nih.gov/pubmed/?term=Pereira%20DIA%5BAuthor%5D&cauthor=true&cauthor_uid=30569038), [Mohammed NI](https://www.ncbi.nlm.nih.gov/pubmed/?term=Mohammed%20NI%5BAuthor%5D&cauthor=true&cauthor_uid=30569038), [Ofordile O](https://www.ncbi.nlm.nih.gov/pubmed/?term=Ofordile%20O%5BAuthor%5D&cauthor=true&cauthor_uid=30569038), [Camara F](https://www.ncbi.nlm.nih.gov/pubmed/?term=Camara%20F%5BAuthor%5D&cauthor=true&cauthor_uid=30569038), [Baldeh B](https://www.ncbi.nlm.nih.gov/pubmed/?term=Baldeh%20B%5BAuthor%5D&cauthor=true&cauthor_uid=30569038), [Mendy T](https://www.ncbi.nlm.nih.gov/pubmed/?term=Mendy%20T%5BAuthor%5D&cauthor=true&cauthor_uid=30569038), et al. A novel nano-iron supplement to safely combat iron deficiency and anaemia in young children: The IHAT-GUT double-blind, randomised, placebo-controlled trial protocol. [Gates Open Res.](https://www.ncbi.nlm.nih.gov/pubmed/30569038) 2018 Oct 11;2:48. doi: 10.12688/gatesopenres.12866.2.
4. [Shim JY](https://www.ncbi.nlm.nih.gov/pubmed/?term=Shim%20JY%5BAuthor%5D&cauthor=true&cauthor_uid=30153811), [Kim MY](https://www.ncbi.nlm.nih.gov/pubmed/?term=Kim%20MY%5BAuthor%5D&cauthor=true&cauthor_uid=30153811), [Kim YJ](https://www.ncbi.nlm.nih.gov/pubmed/?term=Kim%20YJ%5BAuthor%5D&cauthor=true&cauthor_uid=30153811), [Lee Y](https://www.ncbi.nlm.nih.gov/pubmed/?term=Lee%20Y%5BAuthor%5D&cauthor=true&cauthor_uid=30153811), [Lee JJ](https://www.ncbi.nlm.nih.gov/pubmed/?term=Lee%20JJ%5BAuthor%5D&cauthor=true&cauthor_uid=30153811), [Jun JK](https://www.ncbi.nlm.nih.gov/pubmed/?term=Jun%20JK%5BAuthor%5D&cauthor=true&cauthor_uid=30153811), et al. Efficacy and safety of ferric carboxymaltose versus ferrous sulfate for iron deficiency anemiaduring pregnancy: subgroup analysis of Korean women. [BMC Pregnancy Childbirth.](https://www.ncbi.nlm.nih.gov/pubmed/?term=Efficacy+and+safety+of+ferric+carboxymaltose+versus+ferrous+sulfate+for+iron+deficiency+anemia+during+pregnancy%3A+subgroup+analysis+of+Korean+women) 2018 Aug 28;18(1):349. doi: 10.1186/s12884-018-1817-y.
5. [Breymann C](https://www.ncbi.nlm.nih.gov/pubmed/?term=Breymann%20C%5BAuthor%5D&cauthor=true&cauthor_uid=27278921), [Milman N](https://www.ncbi.nlm.nih.gov/pubmed/?term=Milman%20N%5BAuthor%5D&cauthor=true&cauthor_uid=27278921), [Mezzacasa A](https://www.ncbi.nlm.nih.gov/pubmed/?term=Mezzacasa%20A%5BAuthor%5D&cauthor=true&cauthor_uid=27278921), [Bernard R](https://www.ncbi.nlm.nih.gov/pubmed/?term=Bernard%20R%5BAuthor%5D&cauthor=true&cauthor_uid=27278921), [Dudenhausen J](https://www.ncbi.nlm.nih.gov/pubmed/?term=Dudenhausen%20J%5BAuthor%5D&cauthor=true&cauthor_uid=27278921); [FER-ASAP investigators](https://www.ncbi.nlm.nih.gov/pubmed/?term=FER-ASAP%20investigators%5BCorporate%20Author%5D). Ferric carboxymaltose vs. oral iron in the treatment of pregnant women with iron deficiency anemia: an international, open-label, randomized controlled trial (FER-ASAP). [J Perinat Med.](https://www.ncbi.nlm.nih.gov/pubmed/27278921) 2017 May 24;45(4):443-453. doi: 10.1515/jpm-2016-0050.
6. [Khalafallah AA](https://www.ncbi.nlm.nih.gov/pubmed/?term=Khalafallah%20AA%5BAuthor%5D&cauthor=true&cauthor_uid=30502851), [Hyppa A](https://www.ncbi.nlm.nih.gov/pubmed/?term=Hyppa%20A%5BAuthor%5D&cauthor=true&cauthor_uid=30502851), [Chuang A](https://www.ncbi.nlm.nih.gov/pubmed/?term=Chuang%20A%5BAuthor%5D&cauthor=true&cauthor_uid=30502851), [Hanna F](https://www.ncbi.nlm.nih.gov/pubmed/?term=Hanna%20F%5BAuthor%5D&cauthor=true&cauthor_uid=30502851), [Wilson E](https://www.ncbi.nlm.nih.gov/pubmed/?term=Wilson%20E%5BAuthor%5D&cauthor=true&cauthor_uid=30502851), [Kwok C](https://www.ncbi.nlm.nih.gov/pubmed/?term=Kwok%20C%5BAuthor%5D&cauthor=true&cauthor_uid=30502851), et al. A Prospective Randomised Controlled Trial of a Single Intravenous Infusion of Ferric Carboxymaltose vs Single Intravenous Iron Polymaltose or Daily Oral Ferrous Sulphate in the Treatment of Iron Deficiency Anaemia in Pregnancy. [Semin Hematol.](https://www.ncbi.nlm.nih.gov/pubmed/30502851) 2018 Oct;55(4):223-234. doi: 10.1053/j.seminhematol.2018.04.006. Epub 2018 Apr 25.
7. Ugwu EO, Olibe AO, Obi SN, Ugwu AO. Determinants of compliance to iron supplementation among pregnant women in Enugu, Southeastern Nigeria. Niger J Clin Pract 2014; 17: 608-12.
8. Eleje GU, Obiagwu HI, Ogbuokiri AC, Ilika CP, Osuagwu EP. Successful management of severe anemia without blood transfusion in pregnancy and puerperium using Mojeaga® as adjunct therapy: A report of two cases. Hong Kong J Obst Gynae 2018; 1(1): 01-03
9. Peter. Mojega herbal remedy and treasure herbs are a 100% natural herbal. Available at:https://www.abuja-ng.com/mojega-herbal-remedy-and-treasure-herbs-are-a-100-natural-herbal-08130122561-abujalagos.html. Assessed on 23rd December, 2018.
10. Herbal Remedy Rescue Sickle Cell Sufferers In Edo. Available at: <http://www.thenigerianpost.com.ng/herbal-remedy-rescue-sickle-cell-sufferers-in-edo/>. Assessed on 23rd December, 2018.
11. New herbal remedy offers hope for sickle cell sufferers. Available at:https://globalpatriotnews.com/new-herbal-remedy-offers-hope-for-sickle-cell-sufferers/. Assessed on 23rd December, 2018.

**APPENDIX 1: CONSENT FORM TO PARTICIPATE IN A RESEARCH STUDY**

The purpose of this consent form is to tell you the information you need to know in deciding whether to participate in this research study entitled: **Efficacy and safety of Mojeaga^®^ in combination with conventional oral iron therapy for correcting anemia in gynecological and obstetrics patients: a pilot randomized clinical trial in NAUTH, Nnewi.**

**STUDY PURPOSE**

You are invited to participate in a research study. You are under no obligation to participate and if you decide not to participate it will not affect your hospital care in any way.

Anemia (shortage of blood) is a serious problem. Persons with anemia at high risk of deaths and their babies.

As at now, there are some drugs approved for use to correct anemia such as astyfer and fesolate, certain hematinics and blood transfusion. Other local food supplement like Mojeaga has been used with some success.

There's still much to learn. We don't really understand to what extent this Mojeaga works, even though it strongly claimed to be very good for treatment of anemia. So the use of the drug may yield more hope that is equivalent to the drugs already in use.

**STUDY PROCEDURE**

The study will include adult patients suffering from anemia. If you decide to participate in this study, you will undergo the regular check by your doctor, which includes a general exam and collection of blood sample to confirm that you really have the anemia. So the performance of the medical exam and collection of blood sample will be done during routine standard assessment. There will be no additional discomfort to you.

**STUDY RISKS**

The potential risks with participating in this study include nausea, and vomiting on the patients. However, to overcome these risks, patients must be mornitored, very lowest effective dose will be used for the study. Billions of people ingest it daily, and no government had advised that it is dangerous. However, any patient found to be developing complications such as fainting following recruitment will be discontinued from the study and be given appropriate treatment free of charge.

# POTENTIAL BENEFITS

**BENEFICIENCE:** The immediate benefits include defraying the cost of test used in the diagnosis of anemia. The expected benefit of this study to the medical community and patients is the availability of a simpler and cheaper drug for treating patients with anemia. Hopefully with findings in this study, Mojeaga might well become the one of the standard drug for treating anemia in the world.

**PARTICIPATION IS VOLUNTARY**

Your participation in this study is completely voluntary. Whether or not you participate in this study, you will still get the standard medical treatment for your condition. You can withdraw from the study at any time, and such a decision will not affect your medical care

**CONFIDENTIALITY**

Any information obtained during this study and discussed with you will be anonymously published publicly.

# QUESTIONS

# If you have any questions, please ask and we will do our best to answer them.

I, Mrs/Miss --------------------------------------------------------------------------------------------------do hereby give consent/permission to be included in the intended research as explained to me in English or Igbo Language and understood by me.

I have been made to understand that my participation to the study is voluntary and if I withdraw from the study, I will still enjoy the same standard of care given to any other patients by the doctor without prejudice.

SIGNATURE OF PARTICIPANT_______________________DATE___________

WITNESSS----------------------------------------------------------DATE------------------

NAME OF RESEARCHERS—**DR ELEJE GEORGE UCHENNA, PROF. JOSEPH IFEANYICHUKWU IKECHEBELU, PROF. CHARLES .O. ESIMONE, DR IC AJUBA, CHIKA ASUOHA-------------------------**PHONE NUMBER OF RESEARCHER—**08068117444**--------------------------------------

**PROFORMA: Efficacy and safety of Mojeaga^®^ in combination with conventional oral iron therapy for correcting anemia in gynecological and obstetrics patients: a pilot randomized clinical trial in NAUTH, Nnewi.**

**NAME OF DOCTOR…………………………………………CODE NUMBER OF PATIENT……………………….…………….**

1. COMPUTER NUMBER OF PATIENT……………………………………… ...…..(2) GENDER………………………
2. DATE OF RECRUITMENT……………….... (4) TIME……………… (5) AGE……………...…………………

6.) Marital status : Married [ ] [single [ ] Divorced [ ](7.) LMP------------------ (8) GESTATIONAL AGE----------------

8. Parity…………….. 9. Body weight-------------- 9. Height: (cm)………………………………………………

**TICK[X] OR WRITE WHICHEVER IS APPROPRIATE.**

1. **RETROVIRAL DISEASE STATUS**
2. Negative [ ] (b) Positive [ ]
3. HIGHEST EDU LEVEL…………………………………12.) OCCUPATION (Specify)…………………………….………………….

13. PREVIOUS Chronic Anemia? a.) YES----b.) NO------ (15B)Known Hypertensive Yes [ ], NO [ ]

16. MARITAL STATUS

1. Single [ ] (b) Married [ ]

17. FINDINGS AT CLINICAL EXAMINATION before therapy…………?**TICK [√] below:**

| **VARIABLE before therapy** | **PRESENCE** | **ABSENCE** |
| --- | --- | --- |
| Epigastrric pain |  |  |
| Nausea |  |  |
| Vomiting |  |  |
| Dizziness |  |  |
| Weakness |  |  |
| Hematemesis |  |  |

18. FINDINGS AT CLINICAL EXAMINATION AFTER therapy……………?**TICK [√] below:**

| **VARIABLE AFTER therapy** | **PRESENCE** | **ABSENCE** |
| --- | --- | --- |
| Epigastrric pain |  |  |
| Nausea |  |  |
| Vomiting |  |  |
| Dizziness |  |  |
| Weakness |  |  |
| Hematemesis |  |  |

**19. SOME LABORATORY PARAMETERS**

| **LABORATORY VARIABLE** | **VALUES BEFORE THERAPY** | **VALUES AFTER THERAPY** | **NORMAL RANGES** |
| --- | --- | --- | --- |
| Na |  |  |  |
| K |  |  |  |
| Bicarbonate |  |  |  |
|  |  |  |  |
| AST |  |  |  |
| ALP |  |  |  |
| ALT |  |  |  |
|  |  |  |  |
| PCV/hematocrit |  |  |  |
| Hb |  |  |  |
| Wbc |  |  |  |
| Platelets |  |  |  |
|  |  |  |  |
|  |  |  |  |
|  |  |  |  |
|  |  |  |  |

**FETAL/NEONATAL OUTCOME during therapy**

1. **Preterm labour** a.) YES----b.) NO------
2. **Preterm PROM** a.) YES----b.) NO------
3. **Birth weight (kg)----------------------------**
4. **Birth anomaly---------------**

**PROFORMA: Efficacy and safety of Mojeaga® in combination with conventional oral iron therapy for correcting anemia in gynecological and obstetrics patients: a pilot randomized clinical trial in NAUTH, Nnewi.**

**NAME OF DOCTOR................................................CODE NUMBER OF PATIENT………………………**

(1). COMPUTER NUMBER OF PATIENT..........................(2) GENDER…………………………

(3). DATE OF RECRUITMENT......................(4) TIME..................(5) AGE.......................

(6.) Marital status: Married [ ] [single [ ] Divorced [ ](7.) LMP………..(8)GESTATIONAL AGE..............

(9). Parity.................(10). Body weight-.............(11). Height: (cm)......................................................

**TICK [X] OR WRITE WHICHEVER IS APPROPRIATE.**

**(12) RETROVIRAL DISEASE STATUS**

a.) Negative [ ] (b) Positive [ ]

(14). HIGHEST EDU LEVEL.................................(15.) OCCUPATION (Specify)..............................

(16). PREVIOUS Chronic Anemia? (a.) YES—-(b.) NO----- (16B) Known Hypertensive Yes [ ], NO [ ]

(17). MARITAL STATUS

a.) Single [ ] (b) Married [ ]

(18). FINDINGS AT CLINICAL EXAMINATION BEFORE AND AFTER therapy.......**? TICK [√] below:**

| **VARIABLE BEFORE therapy** | **PRESENCE** | **ABSENCE** | **VARIABLE AFTER therapy** | **PRESENCE** | **ABSENCE** |
| --- | --- | --- | --- | --- | --- |
| Epigastric pain |  |  | Epigastric pain |  |  |
| Nausea |  |  | Nausea |  |  |
| Vomiting |  |  | Vomiting |  |  |
| Dizziness |  |  | Dizziness |  |  |
| Weakness |  |  | Weakness |  |  |
| Hematemesis |  |  | Hematemesis |  |  |

**19. SOME LABORATORY PARAMETERS**

| **LABORATORY VARIABLE** | **VALUES BEFORE THERAPY** | **VALUES AFTER THERAPY** | **NORMAL RANGES** |
| --- | --- | --- | --- |
| **Na** |  |  |  |
| **K** |  |  |  |
| **Bicarbonate** |  |  |  |
|  |  |  |  |
| **AST** |  |  |  |
| **ALP** |  |  |  |
| **ALT** |  |  |  |
|  |  |  |  |
| **PCV/hematocrit** |  |  |  |
| **Hb** |  |  |  |
| **Wbc** |  |  |  |
| **Platelets** |  |  |  |
|  |  |  |  |
|  |  |  |  |
|  |  |  |  |
|  |  |  |  |

**FETAL/NEONATAL OUTCOME during therapy**

1. Preterm labour a.) YES—-b.) NO—-

2. Preterm PROM a.) YES—b.)NO-----

3. Birth weight (kg)----------------------------

4. birth anomaly
