# Supplementary material for: Efficacy and safety of Mojeaga remedy in combination with conventional oral iron therapy for correcting anemia in obstetric population: A phase II randomized pilot clinical trial
Source: PLoS One. 2023 May 8;18(5):e0285474. doi: 10.1371/journal.pone.0285474 (PMC10166496; doi:10.1371/journal.pone.0285474)
Supplement: Attachment — Submitted filename: PONE.docx [file pone.0285474.s006.docx]

PONE-D-23-04244

Efficacy and safety of Mojeaga remedy in combination with conventional oral iron therapy for correcting anemia in obstetric population: a phase II randomized pilot clinical trial

Dr. GEORGE UCHENNA ELEJE

PLOS ONE

Some comments

*P9: There was no comparable groups in this study receiving different doses of the intervention therapy to detect as mentioned potentially increase side effects due to excess unabsorbed iron remaining in the GIT

This is out of the scope of the study to be mentioned

* The randomization was done by the study site (didn’t mention the number (%) taken from each strata in case strata differed in overall health or in exposure to risk factors of anemia

*Control therapy P13-14: The frequency of daily intake is to be revised; two or three times daily (unclear)

* Secondary outcome P15: spelling mistake ; epigatric pain- epigastric pain

* Sampling approach P16: No sampling should be done for anemic patients before randomization

Could be written as such: All individuals during the period from …. to …. after satisfying the inclusion and the exclusion criteria were randomly allocated.

*Results: Mean age of the study groups was not mentioned

*P33: Through Thorough ???

*P 35 discussion: efficacy and effectiveness

*Monitoring of the liver and the renal functions. Does a period of two weeks is sufficient for them to change ?References are needed for confirmation
