# Supplementary material for: Efficacy and safety of Mojeaga remedy in combination with conventional oral iron therapy for correcting anemia in obstetric population: A phase II randomized pilot clinical trial
Source: PLoS One. 2023 May 8;18(5):e0285474. doi: 10.1371/journal.pone.0285474 (PMC10166496; doi:10.1371/journal.pone.0285474)
Supplement: Attachment — Submitted filename: REPLY-MOJEAGA REVIEWER COMMENTS DEC 2022.docx [file pone.0285474.s004.docx]

2-11-2023

From

Corresponding author (George Eleje)

To

Editor

***PLOS ONE***
Dear editors:

**Re: Submission of Response to Reviewers’ comments on Manuscript ID** PONE-D-22-25881 titled: ‘***Efficacy and safety of Mojeaga remedy in combination with conventional oral iron therapy for correcting anemia in obstetric population: a phase II randomized pilot clinical trial***.’

We want to express our gratitude to our Reviewer and editorial team for adding so much value to our work. We have now responded adequately. Please find enclosed a point-by-point response to the comments by the editors. We hope that the editors and reviewers will find the revisions acceptable.

**REVIEWER COMMENT**

The reviewers have expressed concerns that sufficient justification of exposing people to a new intervention appears to be lacking. In addition, the reviewers indicate that there are concerns with the reporting of the sample size calculation and the statistical analysis. In addition, the reviewers have raised concerns with the robustness of the study and the assessment of the effects, and the reporting of the sampling methodology.

In light of the concerns identified, the manuscript does not meet PLOS ONE’s criteria for publication in its current form.

If, at a later stage, you are able to revise the manuscript to address the concerns raised by the reviewers, you may wish to consider submitting a revised manuscript to PLOS ONE. This would however require a substantial amount of work and thus, would be considered as a new submission.

I am sorry that we cannot be more positive on this occasion, but I do hope that you will find these comments useful when deciding how to proceed with your manuscript.

Kind regards,

Maria Elisabeth Johanna Zalm, Ph.D
Editorial Office
PLOS ONE

AUTHORS’ RESPONSE

Many thanks for giving us the opportunity to revise the manuscript.

We have responded to all issues adequately.

**REVIEWER COMMENT**

Additional Editor Comments (if provided):

Reviewers' comments:

Reviewer's Responses to Questions

**Comments to the Author**

1. Is the manuscript technically sound, and do the data support the conclusions?

The manuscript must describe a technically sound piece of scientific research with data that supports the conclusions. Experiments must have been conducted rigorously, with appropriate controls, replication, and sample sizes. The conclusions must be drawn appropriately based on the data presented.

Reviewer #1: Yes

Reviewer #2: Partly

2. Has the statistical analysis been performed appropriately and rigorously?

Reviewer #1: Yes

Reviewer #2: No

3. Have the authors made all data underlying the findings in their manuscript fully available?

The [PLOS Data policy](http://www.plosone.org/static/policies.action#sharing) requires authors to make all data underlying the findings described in their manuscript fully available without restriction, with rare exception (please refer to the Data Availability Statement in the manuscript PDF file). The data should be provided as part of the manuscript or its supporting information, or deposited to a public repository. For example, in addition to summary statistics, the data points behind means, medians and variance measures should be available. If there are restrictions on publicly sharing data—e.g. participant privacy or use of data from a third party—those must be specified.

Reviewer #1: Yes

Reviewer #2: Yes

4. Is the manuscript presented in an intelligible fashion and written in standard English?

PLOS ONE does not copyedit accepted manuscripts, so the language in submitted articles must be clear, correct, and unambiguous. Any typographical or grammatical errors should be corrected at revision, so please note any specific errors here.

Reviewer #1: Yes

Reviewer #2: No

5. Review Comments to the Author

Please use the space provided to explain your answers to the questions above. You may also include additional comments for the author, including concerns about dual publication, research ethics, or publication ethics. (Please upload your review as an attachment if it exceeds 20,000 characters)

AUTHORS’ RESPONSE

We have responded to all issues adequately.

**REVIEWER COMMENT**

Reviewer #1: Efficacy and safety of Mojeaga remedy in combination with conventional oral iron therapy for correcting anemia in obstetric population: a phase II randomized pilot clinical trial

1. Abstract; absence of clinical trial is not a sufficient justification of exposing human participants to new intervention. Authors must provide adequate justification on the gap and its relevance

AUTHOR REPLY

A recent systematic review and meta-analysis involving 15 eligible studies with a total of 1938 participants concluded that there is no strong evidence that first-line therapy with intravenous iron is superior to oral administration for treating IDA in pregnant women [1]. The few identified differences in outcomes were small in magnitude and from studies at high risk of bias.

Mojeaga herbal remedy (produced by Mojeaga International Ventures Ltd, Nigeria) is a natural preparation containing a combined *Alchornea cordifolia, Pennisetum glaucum,* and *Sorghum bicolor* extracts*. Alchornea cordifolia, Pennisetum glaucum,* and *Sorghum bicolor* have been used for centuries due to its medicinal properties and health benefits.  Therapy with Mojeaga herbal remedy has emerged as one of the adjunct to conventional therapy for the treatment of anemia in obstetric population. It is uncertain the benefits and safety of the therapy in the obstetric population, and whether the two weeks’ duration of therapy should suffice for clinical practice. It is also uncertain if higher combination doses will potentially increase side effects due to the excess unabsorbed iron remaining in the gastrointestinal tract. According to current UK guidelines on the management of iron deficiency in pregnancy by Pavord et al., it was revealed that for anemic women, a trial of oral iron should be considered as the first line diagnostic test, whereby an increment demonstrated at two weeks is a positive result [2, 3]. Parenteral iron should be considered for women with confirmed iron deficiency who fail to respond to or are intolerant of oral iron. The anecdotal report and recent case report have revealed that combined mojeaga remedy and conventional iron therapy may be superior to conventional oral iron alone for treatment of iron-deficiency anemia in pregnancy. However, this has not been demonstrated in randomized trials. A recent trial on combination iron therapy involving iron sucrose and oral iron bisglycinate recommended that future studies should focus on other oral iron supplements to find the most efficient treatment protocol with regard to red cell increase and adverse effects rate [4]. It is therefore uncertain whether women receiving combined mojeaga remedy and conventional iron therapy more often achieve desired hematocrit targets, faster and with fewer side effects. It is also unknown whether their combination will further improve hematocrit level or will cause adverse effects without hematological or functional benefit. We therefore aimed to assess the efficacy, safety and tolerability of Mojeaga as adjunct to conventional oral iron therapy for correction of anemia in obstetric population.

References

1. Qassim A, Grivell RM, Henry A, Kidson-Gerber G, Shand A, Grzeskowiak LE. Intravenous or oral iron for treating iron deficiency anaemia during pregnancy: systematic review and meta-analysis. Med J Aust. 2019 Oct;211(8):367-373. doi: 10.5694/mja2.50308.
2. Pavord S, Daru J, Prasannan N, Robinson S, Stanworth S, Girling J, et al. UK guidelines on the management of iron deficiency in pregnancy. Br J Haematol. 2020 Mar;188(6):819-830. doi: 10.1111/bjh.16221.
3. Pavord S, Myers B, Robinson S, Allard S, Strong J, Oppenheimer C, et al. UK guidelines on Yefet E, Mruat Rabah S, Sela ND, Hosary Mhamed S, Yossef A, Nachum Z. Addition of oral iron bisglycinate to intravenous iron sucrose for the treatment of postpartum anemia-randomized controlled trial. Am J Obstet Gynecol. 2021 Dec;225(6):668.e1-668.e9. doi: 10.1016/j.ajog.2021.06.069.
4. the management of iron deficiency in pregnancy. Br J Haematol. 2012 Mar;156(5):588-600. doi: 10.1111/j.1365-2141.2011.09012.x. Erratum in: Br J Haematol. 2012 Aug;158(4):559.

**REVIEWER COMMENT**

2. Plant names are incorrectly identified. When introducing plant names for the first, should be identified with correct binomial names, including authority and family. Their subsequent use could follow their standard short form. Authors could observe this practice throughout the manuscript

AUTHORS’ RESPONSE

Many thanks for pinpointing the error in names of plant. We have corrected and amended as follows in the manuscript. Mojeaga herbal remedy (produced by Mojeaga International Ventures Ltd, Nigeria) is a natural preparation containing a combined *Alchornea cordifolia, Pennisetum glaucum,* and *Sorghum bicolor* extracts

**REVIEWER COMMENT**

3. The statements which are not facts should be properly cited. For instance the statement “It increases the levels of packed cell volume and hemoglobin and contains antioxidants and natural minerals” requires citation.

AUTHORS’ RESPONSE

We have added the citation as recommended. The sentence is as follows: It increases the levels of packed cell volume and hemoglobin levels and contains antioxidants and natural minerals [8, 9].

**REVIEWER COMMENT**

4. A follow up of two weeks is not sufficient to assess the efficacy of intervention of anemia. Authors could provide justification on such shorter time in relation to established optimal time for adequate recovery from anemia

AUTHORS’ RESPONSE

Many thanks for the request for further clarifications on the use of two weeks assessment criteria period. In the study, we adopted the two weeks assessment for efficacy of intervention of anemia because, a prior case report reported a two week therapy. This was also the protocol approved by the ethics committee, Pan African Clinical Trial registry and NAFDAC, a Nigerian monitoring body for clinical trials of new drugs. Additionally, According to current UK guidelines on the management of iron deficiency in pregnancy by Pavord et al., it was revealed that for anemic women, a trial of oral iron should be considered as the first line diagnostic test, whereby an increment demonstrated at two weeks is a positive result, provided that oral iron is taken according to the recommendations. The UK current guideline further revealed that a trial of iron therapy for simultaneous diagnostic and therapeutic purposes is helpful, and a rise in hemoglobin level should be demonstrable by 2 weeks which supports the diagnosis of iron deficiency. According to the guideline, if there has been no improvement in hematocrit level following 2 weeks of optimal therapy and compliance, more definitive testing and treatment is required. The guideline by Pavord et al further stated that **if anemia without an obvious other cause is detected, a diagnostic trial of oral iron should be given without delay, with a repeat full blood count in 2–3 weeks (1D).**

**Addition, our protocol of assessing efficacy of intervention of anaemia in two weeks was similar to a French study by** Broche **et al iron use in Pregnancy[3] [**Broche DE, Gay C, Armand-Branger S, Grangeasse L, Terzibachian JJ. Anémies sévères du post-partum immédiat. Pratique clinique et intérêt du fer par voie intraveineuse [Acute postpartum anaemia. Clinical practice and interest of intravenous iron]. Gynecol Obstet Fertil. 2004 Jul-Aug;32(7-8):613-9. French. doi: 10.1016/j.gyobfe.2004.05.014.]

**However, the** optimal time for adequate recovery can be achieved with iron supplements and can depend on the hematocrit and iron status at the start of supplementation, ongoing losses, iron absorption and other factors contributing to anemia, such as other micronutrient deficiencies, infections and renal impairment. However, compliance and intolerance of oral iron preparations are the usual factors limiting efficacy. These confounders were ensured that they were removed in the trial. According to the UK guideline, a repeat blood cell level at 2–3 weeks is required to assess response to treatment **[1, 2].**. According to the guideline, **repeat hemogram testing is required 2–3 weeks after commencing treatment for established anemia, to assess compliance, correct administration and response to treatment (1B) [1, 2].**

Finally, this time might not be enough to completely recover. However, it is sufficient to clinically assess the improvement in anemia based on the previous results and the disappearance of anaemic symptoms.

1. Pavord S, Daru J, Prasannan N, Robinson S, Stanworth S, Girling J, et al. UK guidelines on the management of iron deficiency in pregnancy. Br J Haematol. 2020 Mar;188(6):819-830. doi: 10.1111/bjh.16221.
2. Pavord S, Myers B, Robinson S, Allard S, Strong J, Oppenheimer C, et al. UK guidelines on the management of iron deficiency in pregnancy. Br J Haematol. 2012 Mar;156(5):588-600. doi: 10.1111/j.1365-2141.2011.09012.x. Erratum in: Br J Haematol. 2012 Aug;158(4):559.
3. Broche DE, Gay C, Armand-Branger S, Grangeasse L, Terzibachian JJ. Anémies sévères du post-partum immédiat. Pratique clinique et intérêt du fer par voie intraveineuse [Acute postpartum anaemia. Clinical practice and interest of intravenous iron]. Gynecol Obstet Fertil. 2004 Jul-Aug;32(7-8):613-9. French. doi: 10.1016/j.gyobfe.2004.05.014.

**REVIEWER COMMENT**

5. Change the word “effectiveness” to “efficacy”

AUTHORS’ RESPONSE

Many thanks for the suggestions. We have changed all to efficacy.

**REVIEWER COMMENT**

6. Worms and protozoan diseases such as malaria also causes anemia, how this was controlled in the design and analysis of the data. This is not well discussed

AUTHORS’ RESPONSE

Many thanks for the suggestions. All patients received intermittent treatment for malaria. However, the protocol did not involve routine use of antihelminthic therapy such as mebendazole. We have now appended as one of the limitations of the study as follows. This study did not control for worms or helminthic diseases which could be attributable as one of the causes of anemia in pregnancy, as the participants did not routinely receive antihelminthic therapy such as mebendazole during pregnancy.

**REVIEWER COMMENT**

7. Provide sufficient information regarding “standard of care” what constituted standard of care?

“Standard of care” constituted: Standard doses of one capsule of Astyfer (a supplement with Iron, Amino Acids and Vitamin B complex; Til Healthcare PVT Ltd, Andhra Pradesh, 517588 India) and tablet vitamin C 100mg 3 times daily administered two times a day (breakfast and dinner) for 2 weeks.
We have appended as follows under methodology as follows: The standard-of-care consist of standard doses of one capsule of Astyfer (a supplement with Iron, Amino Acids and Vitamin B complex; Til Healthcare PVT Ltd, Andhra Pradesh, 517588 India) and tablet vitamin C 100mg 3 times daily administered two times a day (breakfast and dinner) for 2 weeks.

**REVIEWER COMMENT**

8. How do hematocrit and hemoglobin relate to each other? What was the basis for selecting the later as a primary endpoint is not well described

AUTHORS’ RESPONSE

We are happy to provide the clarifications on the relationship between hematocrit and hemoglobin. According to Quinto et al, the Hematocrit (%) is usually defined as three times the value of hemoglobin (g/dl), while the clinical definition of anemia is related to either an abnormal hematocrit or Hemoglobin value [1]. The authors of Quinto et al concluded that measurement of hematocrit count is easy and can be performed in most rural health care centres, and the corresponding Hemoglobin levels cannot be derived with an acceptable accuracy using the value 3 as a conversion factor. In contrast, another study in Thailand by Lee et al, involving a large data set concluded that the commonly used threefold conversion is valid and the conversion factor to equate hemoglobin and hematocrit is accurate and robust both in acute uncomplicated malaria and in convalescence cases [2]. Despite these, the both the WHO and the Centers for Disease Control and Prevention (CDC) have continued to relate the hematocrit with 3 times the hemoglobin values. For example, it was documented in a previous study: The World Health Organization (WHO) defines anemia of pregnancy as hemoglobin (Hb) <11 g/dL, or hematocrit <33%, at any time during the pregnancy [3]. The Centers for Disease Control and Prevention (CDC) define anemia of pregnancy as Hb <11 g/dL, or hematocrit <33% during the first and third trimesters [4].

Since this is a pilot study in multi center hospitals located in various regions, we decided to use hematocrit count which was easier to perform and can be performed in most rural health care centres in various settings. In our future phase 3 trial of this study, we plan to utilize hemoglobin marker which ideally should be the measurement of choice for assessing anemia outcomes in intervention trials [5].

We append as follows as one of the limitations of the study: Since this is a pilot study involving multiple hospitals located in various regions, we utilized the hematocrit count as a marker for anemia which was easier to perform in various Nigerian settings. However, in our future phase 3 trial of this study, we plan to utilize hemoglobin marker which ideally should be the measurement of choice for assessing anemia outcomes in intervention trials.

1. Quintó L, Aponte JJ, Menéndez C, Sacarlal J, Aide P, Espasa M, et al. Relationship between haemoglobin and haematocrit in the definition of anaemia. Trop Med Int Health. 2006 Aug;11(8):1295-302. doi: 10.1111/j.1365-3156.2006.01679.x.
2. Lee SJ, Stepniewska K, Anstey N, Ashley E, Barnes K, Binh TQ, et al. The relationship between the haemoglobin concentration and the haematocrit in Plasmodium falciparum malaria. Malar J. 2008 Aug 2;7:149. doi: 10.1186/1475-2875-7-149.
3. World Health Organization. Iron Deficiency Anaemia: Assessment, Prevention and Control: A Guide for Programme Managers. Geneva, Switzerland: World Health Organization; 2001.
4. Centers for Disease Control and Prevention. Recommendations to prevent and control iron deficiency in the United States. *MMWR Recomm Rep.*1998; 47(RR-3):1-29.
5. Carneiro IA, Drakeley CJ, Owusu-Agyei S, Mmbando B, Chandramohan D. Haemoglobin and haematocrit: is the threefold conversion valid for assessing anaemia in malaria-endemic settings? Malar J. 2007 May 22;6:67. doi: 10.1186/1475-2875-6-67.

**REVIEWER COMMENT**

9. Sampling approach is confusing. Further clarification should be provided to qualify approach used in this study as random. To my understanding, this is a typical convenient sampling approach

AUTHORS’ RESPONSE

We are happy to provide the clarifications on the matter of sampling. In Convenience sampling, the participants are consecutively selected in order of appearance according to their convenient. The sampling process comes to an end when the total amount of participants (sample saturation) are reached. We agree with the reviewer that the randomized clinical trials are usually based on convenience sampling. After sampling, participants are usually randomly allocated to the intervention or control group (randomization). We therefore append as follows: The randomized clinical trials are usually based on convenience sampling. After sampling, participants were randomly allocated to the intervention or control group (randomization) of the eligible, consenting women with diagnosis of anemia.

**REVIEWER COMMENT**

10. Although the authors provide certificate of analysis of the product, it is still important to provide adequate information on how the quality of Mojeaga product was assured during its production. Evidence of GMP compliance is critical.

AUTHORS’ RESPONSE

We are happy to provide the clarifications on the issues of certificate of analysis.

The production company has Standard Operating Procedures (SOPs) that that they follow during the production. Additionally, they carry out quality control tests regularly in the laboratory for each batch after production. SOPs were prepared in full compliance with guidelines and organizations and they mirrored current organizational practices. This company has an in-house laboratory where the adequate checks on raw water, fresh leaves, treated water, dried leaves, pH and finished product. The microbiological analysis of raw and treated water, raw materials and finished products are also carried out in in-house laboratory.

**REVIEWER COMMENT**

11. There was a mixed up of follow up time. Some places authors indicated two weeks while in other 12 months. Plz clarify

AUTHORS’ RESPONSE

We are happy to provide the clarifications on the follow up time.

The duration of therapy is two weeks following commencement of therapy while the participants were followed up till delivery of the baby. The issues of the 12 months follow-up has been deleted to avoid confusion for our global readership.

**REVIEWER COMMENT**

Reviewer #2: This manuscript reported results after a pilot randomized control trial studying the efficacy and safety of Mojeaga remedy in combination with conventional oral iron therapy for correcting anemia in obstetric population. This study has several flaws in the design and analysis as listed below:

AUTHORS’ RESPONSE

We are happy to provide the clarifications on the flaws discovered.

**REVIEWER COMMENT**

1. The safety outcomes included both maternal and neonatal outcomes. However, there are 10 out of 95 enrolled women who were puerperal. Therefore, these 10 women's neonatal outcomes should be before taking Mojeaga. It is confusing why these 10 women were also included in the safety comparison.

AUTHORS’ RESPONSE

We are happy to provide the clarifications on the maternal and neontatal outcomes. In the results section, we appended as follows: Of the 95 participants, 85 were pregnant: Mojeaga group (n=42) and standard-of-care group (n=43), while 10 were puerperal women (Mojeaga group (n=6) and standard-of-care group (n=4). So far, all the 95 participants completed the treatment and were included in analysis. As shown in Table 5, the fetal safety outcomes were only analysed for only 85 pregnant women.

**REVIEWER COMMENT**

2. There are three sites involved in this study. It is unclear if there is a site difference from any analysis or if the randomization was stratified by site.

AUTHORS’ RESPONSE

We are happy to provide the clarifications on the site differences. We append as follows: The randomization **was stratified by study site.**

**REVIEWER COMMENT**

3. The treatment lasts for 2 weeks. It is unclear how many patients had missing post treatment measurements as it was only mentioned in the statistical analysis part that "Participants with no observed outcome were considered as treatment failures." However, the primary endpoint is hematocrit level which is a numerical value, not treatment failure or not.

AUTHORS’ RESPONSE

We are happy to provide the clarifications on the treatment failures. There were no cases of treatment failure. We append as follows: There were no cases of treatment failure.

**REVIEWER COMMENT**

4. The randomization part seems to only use simple randomization, which is different than block randomization. If block randomization was used indeed, the block sizes used should be reported.

AUTHORS’ RESPONSE

We are happy to provide the clarifications on the use of the word simple randomization. Actually block randomization was used. We used blocks of 4.

**REVIEWER COMMENT**

5. It was mentioned in the statistical analysis part that an interim analysis about principal efficacy was performed. However, this study was not designed as a group sequential design. Therefore, the overall type I error resultant from this formal interim analysis of primary endpoint may be inflated. In addition, such interim analysis results were not reported in the manuscript.

AUTHORS’ RESPONSE

We are happy to provide the clarifications on the

We appreciate the viewpoint of the reviewer. Group sequential design is **an example of a statistical approach in clinical trial design**. It means that the sample size of the trial is not fixed in advance, and data is sequentially evaluated as it is collected. This is known as interim analysis, and might be carried out at several points in time. A **group sequential design** is a type of [adaptive design](https://www.statisticshowto.com/adaptive-design/) where the number of patients is not set in advance. Patients are divided into an equal number of groups and data is analyzed at pre-determined points in the trial. A **stopping rule** specifies when and why a trial might be halted at these points.

In our trial, the protocol prespecified one interim analyses that allowed for early stopping for efficacy when about 50% of the total expected outcomes in the study had been observed. We included this interim analysis to ensure that we could quickly obtain a positive result if the benefit of Mojeaga was large enough to allow detection well before the maximum sample size (47) was reached. However, in case the benefit of Mojeaga was more modest, we wanted the trial to include enough participants to identify a smaller yet clinically important improvement over standard of care. We believe that every time the data is analysed, there is a 5% chance of making a Type I error, if α=0.05. As the number of analyses increase, the chance of making a Type I error also increases. As a result, an alpha adjustment will often be required. However, since we had only one interim analysis, the overall type I error resultant from this formal interim analysis of primary endpoint was not inflated as we eventually used p<0.05 as marker for test of significance in the study.

The result of the interim analysis is as follows:

Forty women were randomized to receive either combined Mojeaga or standard of care (n=20) or standard of care alone (n=20). Baseline characteristics of the participants were similar between the two groups. Mean rise in hematocrit values at 2 weeks follow-up was significantly higher in Mojeaga group (9.19±5.29% vs 5.70±1.75%; p=0.008). Mean hematocrit values at 2 weeks follow-up was significantly higher in Mojeaga group (30.05±5.29% vs 27.90±2.08%;p<0.001). No difference was seen in tolerability, maternal side effects, renal and hepatic function test parameters (p>0.05). There were no serious adverse advents and no incidence of maternal or fetal death.

**REVIEWER COMMENT**

6. Simple random sampling method in statistics refers to how to select obstetric women to enroll in this study, not just the randomization part after study participants were enrolled in this study. This needs to be corrected in the manuscript.

AUTHORS’ RESPONSE

We are happy to provide the clarifications on the simple randomization issues. We totally agree that simple random sampling method in statistics refers to how to select obstetric women to enroll in this study, not just the randomization part after study participants were enrolled in this study. This has been corrected in the manuscript.

**REVIEWER COMMENT**

7. All tables comparing numerical variables reported U-statistics. To be consistent, median+/-IQR should be reported instead of mean+/-SD.

AUTHORS’ RESPONSE

We are happy to provide the clarifications on the issues raised. As recommended, we have now reported as median+/-IQR instead of mean+/-SD. See Tables 2, 3 and 7

**REVIEWER COMMENT**

8. The sample size calculation was not clear if it was calculated to compare pre-post treatment hematocrit values within each treatment group or compare the change in hematocrit value between two treatment groups.

AUTHORS’ RESPONSE

## We totally understand the perspective of the reviewers. We have corrected and append as follows:

## Since this is a pilot study, we assumed a mean hematocrit level of 29.1% in the control group, mean hematocrit level of 31.5% in the intervention group after the intervention [8], 80% power, 95% confidence interval, standard deviation (SD) of 2.5, and a 15% dropout rate. Based on the assumptions, the minimum sample size required was 94 participants (47 in each arm). Furthermore, this sample size had 97% power to detect a change in hematocrit level from baseline (control: 6.5%; intervention: 10.5; SD: 3.8) between arms.

**REVIEWER COMMENT**

9. There are more than one primary endpoints. But there is no comment about multiple testing adjustment.

AUTHORS’ RESPONSE

We have considered the other primary endpoints, we have appended as follows in the sample size:

## Since this is a pilot study, we assumed a mean hematocrit level of 29.1% in the control group, mean hematocrit level of 31.5% in the intervention group after the intervention [8], 80% power, 95% confidence interval, standard deviation (SD) of 2.5, and a 15% dropout rate. Based on the assumptions, the minimum sample size required was 94 participants (47 in each arm). Furthermore, this sample size had 97% power to detect a change in hematocrit level from baseline (control: 6.5%; intervention: 10.5; SD: 3.8) between arms.
